# The Social Robot in Rehabilitation and Assistance: What Is the Future?

**DOI:** 10.3390/healthcare9030244

**Published:** 2021-02-25

**Authors:** Daniele Giansanti

**Affiliations:** Centre Tisp, Istituto Superiore di Sanità, 00131 Rome, Italy; daniele.giansanti@iss.it; Tel.: +39-06-4990-2701

**Keywords:** e-health, medical devices, m-health, rehabilitation, robotics, organization models, artificial intelligence, electronic surveys, social robots, collaborative robots

## Abstract

This commentary aims to address the field of social robots both in terms of the global situation and research perspectives. It has four polarities. First, it revisits the evolutions in robotics, which, starting from collaborative robotics, has led to the diffusion of social robots. Second, it illustrates the main fields in the employment of social robots in rehabilitation and assistance in the elderly and handicapped and in further emerging sectors. Third, it takes a look at the future directions of the research development both in terms of clinical and technological aspects. Fourth, it discusses the opportunities and limits, starting from the development and clinical use of social robots during the COVID-19 pandemic to the increase of ethical discussion on their use.

## 1. Introduction

We can certainly place among the most marvelous and shocking technological developments of recent years those of collaborative robotics and, among them, those related to social robotics.

The social robot represents an important technological issue to deeply explore both from a technological and clinical point of view. It has been highlighted in an editorial in the Special Issue of the journal Healthcare entitled “Rehabilitation and Robotics: Are They Working Well Together?” [1]. Among the most important directions in the development of social robotics connected to assistance and rehabilitation we find, in a wider approach to the process of rehabilitation and assistance, the following:

- To invest in social robots specifically designed as support during rehabilitation phases (such as, for example, in the care of the elderly).
- To invest in social robots specifically designed as cultural mediators to support during communication/therapy activity (such as in the care of autism).
- To address the problem of empathy in robotics, especially in relation to interaction with social robots.

In fact, starting from the experiences of collaborative robotics, social robots have spread and are opening new opportunities in the field of the rehabilitation and assistance of fragile subjects with different types of problems, ranging from neuromotor disabilities to those of a communicative and psychological type. A particular acceleration in this area has also certainly been due to the COVID-19 pandemic. The need to maintain social distancing, combined with that of (a) ensuring the continuity of care and (b) giving a communicative type of support, has prompted us to look in the direction of social robots as a possible solution at hand: a real lifebuoy. We have, therefore, increasingly begun to look at social robots both, in a more futuristic way, as a potential substitute for human health care and rehabilitation and, in a more realistic and ethically acceptable way, as a reliable possible mediator/facilitator between humans in the field of rehabilitation and assistance. To tell the truth, even before the pandemic, some of the “social” potential of robots had begun to scare us. Recent challenges in some games (which involve a high degree of social interactions based on tactics) between robots and humans have in fact shown us how the computational abilities of robots have definitively knocked out what we previously believed to be the primacy of human intelligence. In 2016, years after Deep Blue’s [2,3,4] famous defeat of Kasparov at chess [5,6], a computer called AlphaGo [7] beat the world champion of *Go* [8,9], a game much more complex than chess; in fact, in this game, the possible options for the first move are 361 (20 in chess) and the second are 130,000 (400 in chess!). According to the scholars of this game, to win, it is necessary to be familiar with the models of social interaction that go far beyond simple computation! The following questions immediately emerge:

- With AlphaGo, are we crossing the threshold between the two forms of artificial and human intelligence, and what does this entail for future developments?
- What is the boundary between a social robot and a powerful computer?
- Does a social robot have at least a mechatronic body (AlphaGo does not have one)?
- Is an interactive video connected to a computer attached to a mobile body/column sufficient to characterize a social robot?
- What degree of autonomy must a social robot have in any case?
- Is all of this ethically acceptable?

As scholars in the field of assistance and rehabilitation, we also question ourselves on these points, which touch on important aspects of (a) scientific research in mechatronics, neuroscience, artificial intelligence and bioengineering; (b) bioethics; and (c) economics and politics, ranging from regulatory to organizational aspects. In light of this, taking into account the focus of this Special Issue, the goal of our study is mainly to produce a commentary that is useful in the field of research without, however, where possible, neglecting the other aspects. In particular, we wish to highlight in this study a map point and a conceptual contextualization of these technologies starting from the roots, which are based on corobotics, and understand what direction these devices are taking and what we can expect in the future.

## 2. The Social Robot as an Evolution of the Collaborative Robot

### 2.1. Collaborative Robots

The term *corobot* or *cobot* derives from the merging of the term collaborative with the term robot [10]. It appeared in the Wall Street Journal in its millennium edition on 1 January 2000 [11] and refers to technologies used since 1996 thanks to the ingenuity of two professors from Northwestern University, J. Edward Colgate and Michael Peshkin. Cobots are robots designed to interact with humans from a certain work environment and in an interaction workspace. Currently, among the robotics sectors, this sector represents one of the greatest developments.

The International Federation of Robotics [12], a professional, nonprofit organization, recognizes two types of robots: industrial robots used in automation and collaborative robots that can be of service for professional and home use. In the field of collaborative robots, there are four groupings:

1. Reactive collaboration: the robot responds to the movement of the worker in real time;
2. Cooperation: the human and robot are both in motion and work simultaneously;
3. Sequential collaboration: the human and robot share part or all of a workspace but do not work simultaneously;
4. Coexistence: there is no shared workspace, but the human and robot work together.

### 2.2. Social Robots

The ability to interact and work with humans is a characteristic of collaborative robots. However, if this interaction and work activity is more characterized by social interaction until it becomes the key role, then we are dealing with a social robot, also called a socially interactive robot [13]. 

In other words, social robots are collaborative robots evolved/specialized in social interaction, and their work is social interaction. 

We must take into account that robots are and will be increasingly part of our lives. Interaction with artificial intelligence in workplaces, shops, healthcare facilities and numerous other meeting places will be increasingly frequent.

Social robots (SRs) in their collaborative interaction are capable [13] of:

- Establishing and maintaining social relationships;
- Learning social skills development and role models;
- Using “natural” signals, such as gestures and gaze;
- Expressing emotions and are able to perceive them;
- Communicating with high-level dialog;
- Expressing one’s own personality and distinctive character.

SRs can be used for a variety of purposes; for example, as educational tools and therapeutic aids. There are several examples of SRs designed for use by elderly people [14,15,16,17], in nursing homes or in hospitals, for example, to:

(a) Support certain motor activities;
(b) Support the elderly during feeding;
(c) Support them in drug therapy; for example, by reminding them to take a drug;
(d) Support them from a cognitive point of view; for example, by stimulating them with games and supporting them from the point of view of communicative interaction, even as simple company;
(e) Or, more generally, provide support as a hospital assistant.

For this reason, SRs are being considered among the key gerontechnologies [17] for the future.

In the COVID-19 era, there has been an increase in the use of SRs in the above-listed desirable activities due to the necessary supervening obligation of social distancing to combat the pandemic [18]. One nonexhaustive example of this is the use of Pepper [7,19] in the UK in this field during the COVID-19 pandemic [20]. Social robotics can also be useful as:

(f) Support in the rehabilitation therapy of communication disabilities such as autism or others, where the robot can represent a useful tool full of stimuli for children [18,21,22,23,24,25,26,27,28].

However, the robots can also be used in the home environment while integrated with home automation technologies by supporting the activities listed above in the elderly. Wakamaru [29], for example, can be integrated into domotics with a wide range of support possibilities. Additionally, so-called home-telepresence robots are headed in this direction. They act as home management mediators/facilitators, allowing communication with other people by means of proper devices (cameras, speakers, microphones, etc.) and improving the subject’s safety. Kuri [30] and JIBO [31] are a family of robots that includes telepresence.

## 3. Research Directions in Social Robots

### 3.1. A Possible Categorization as a Reference

In an interesting review, Sheridan [32] recently categorized the research direction in the field of SRs as follows: (1) Affect, Personality and Adaptation; (2) Sensing and Control for Action; (3) Assistance to the Elderly and Handicapped; (4) Toys and Markets. We summarize this briefly, referring to the review for an in-depth view.

#### 3.1.1. Affect, Personality and Adaptation

The research in this direction [32,33,34,35,36,37,38] concerns using information about the user in order to adapt the SRs to the user’s particular needs and performance intentions, thereby improving acceptance; therefore several studies focus, for example, on how movements of the robot’s body parts imitate human emotions to express different emotions such as anger, disgust, fear, happiness, sadness and surprise.

#### 3.1.2. Sensing and Control for Action

This section considers research that focuses more on the physical interaction between humans and SRs, with consideration to bioengineering solutions [32,39,40,41,42,43,44,45,46,47,48,49,50,51,52,53,54,55,56,57,58,59,60,61,62,63,64,65]. While safety is essential to human–robot collaboration for industrial manipulation and carefully avoiding collisions, in SRs, the guard is different, and great attention is given to the social tasks, such as applying makeup to the human face. More attention has been given to the problem of motion planning, not only for collision avoidance (obviously, safety remains a basic aspect to consider) but also for human likeness. The touch of a robot, in many cases, for example, induces a positive response in a human, so this aspect must be carefully considered.

#### 3.1.3. Assistance to the Elderly and Handicapped

This is one social robot application that has received much attention [32,66,67,68,69,70,71,72,73,74,75,76]. For example, families coping with a relative with autism often struggle with social and emotional communication. In the case of the elderly, the research directions confirm what has been discussed above in Section 1. In the case of the research on the use of robots for children with autism, some gaps have been identified and reported by Sheridan [32], such as diversity in focus, bias in the research toward specific behavior impairments, the effectiveness of the human–robot interaction after impairment and the use of robot-based motor rehabilitation in autism.

#### 3.1.4. Toys and the Market for Social Robots in General

Here, Sheridan [23] makes the important consideration that for user acceptance, government regulator acceptance and sales appeal, engineering/research related to social psychological and human factors should be applied to social robots. This is especially true for children’s toys because children are the most vulnerable of the various user categories. It should be considered that most of the sales of social robots today are for children’s toys as it is possible to see over the web.

### 3.2. Further Personal Considerations

I agree with the categorization identified by Sheridan [32], and I believe that it can be used as a reference for evaluating the future developments of social robots, with particular reference to the assistance and rehabilitation sectors. Without introducing new categorizations and focusing on the rehabilitation sector, I believe that two recent, further considerations are worthy of note. The first is the introduction of a sort of robot-based pet therapy through robots with the appearance of animals. The second is the impact of the research and clinical applications on SRs, as partly anticipated in Section 2 due to the COVID-19 pandemic. Both topics are translational with respect to the four categories described above.

#### 3.2.1. Social-Animal-Like Robot for Pet Therapy

The pet therapy is identified as a complementary intervention that strengthens traditional treatments and can be used on patients suffering from various pathologies, with the aim to improve their state of health, thanks to the human–animal interaction. It has been proved that the presence of an animal (e.g., dog, cat, rubbit) improves both the emotional relationship and the work with the patient, favoring the interaction, attention and in general the communication channel and stimulating the active participation of the subject. Pet therapy is often used in dedicated interventions. 

Pet therapy is now finding fertile ground in SRs. Two examples of this are the two social-animal-like-robots Paro and Robear. Paro was designed by Takanori Shibata in early 1993 [77]. It was designed on the basis of a puppy seal. Paro features a complex mechatronic, with tactile sensors covering its fur, touch-sensitive whiskers and actuators that quietly move its limbs and body. 

Thanks to this design, it responds to cuddles by moving its tail and opening and closing its eyes, memorizes faces, follows the guard and learns actions, generating positive reactions. Among the principal applications [15,16], it is possible to find the same applications of pet therapy in (a) reducing psychological disorders such as anxiety and depression and (b) improving communication skills and (c) the levels of attention and participation. Therefore, the social robot Paro also acts as a rehabilitation therapist. It has been used in rehabilitation therapies on the elderly (for example, with dementia) and on children with autism. Paro is a social companion for those who interact with him, encouraging effects such as increased participation, increased levels of attention and new social performances, such as cooperative attention and interaction [15,16,78,79,80,81,82,83,84,85]. Robear [86] is a white, bear-shaped robot that lifts and helps patients in wheelchairs to move to bed or go to the bathroom. It is a special robot nurse made by the Riken Brain Science Institute [87] that is conquering hospitals in Japan for its efficiency and “sweetness.” Robear is driven by software and three different types of sensors, including “tactile” structures made of rubber. Weighing approximately 140 kg, Robear is strong and agile enough to (a) gently lift the patient from the bed to the wheelchair, (b) help them stand up and (c) move quickly. While the first example, represented by Paro, is a clear example of a pure robot-based pet therapy, the second, Robear, is an example of the application of both robot-based pet therapy and robot-based caregiving, which could also contribute to avoiding caregiver burning during the complex activities of assistance, especially during the COVID-19 pandemic. It should also be considered that many fragile subjects prefer more to be manipulated by a social robot (Robear in this case) than a human caregiver.

#### 3.2.2. Social Robots and COVID-19

The COVID-19 pandemic has dramatically brought to the fore the problem of the frailty of the elderly. Often the elderly were subjected to forced isolation to avoid contagion. This has resulted in both difficulties in health care (including psychological) and the appearance of disturbing factors such as fear, anxiety and other psychological disorders. Their functional capabilities also generally declined during this period. 

To try to minimize the problem, some nursing homes have started using robots to take care of the elderly to try to alleviate their loneliness while supporting them from a mental health point of view. An example of this, as briefly anticipated in Section 2, is the use of Pepper [17] in the UK. SRs, including the previously reported Robear [86], have provided an impetus in research and clinical application during the COVID-19 pandemic. At the end of the pandemic, it will be possible to completely assess this and make a map point.

## 4. Conclusions

The last evolution of collaborative robots (historically proposed for collaboration with human subjects) [10] is the capability to play the role of an interactive social communicator and, therefore, to be a social robot [13]. This new role is showing high potential in both the direction of rehabilitation and assistance of subjects with disabilities, especially the fragile and handicapped. SRs have particularly demonstrated potential both in the care of the elderly and children with communication disabilities, such as autism [9,10,11,12,13,14,15,16,17,18,19,20,21,22]. Recently, we have also witnessed boosted activity both in the research and clinical applications of SRs caused by the COVID-19 pandemic. In fact, SRs present a chance to allow the continuity of care and communication and psychological support in situations where there are rules/initiatives to maintain social distancing to avoid infection; in other terms, a kind of lifebuoy [17,18]. The research direction in the field of SRs has been clearly detected. In an interesting review, Sheridan [32] recently categorized the research direction in this field of SRs as follows: (1) Affect, Personality and Adaptation; (2) Sensing and Control for Action; (3) Assistance to the Elderly and Handicapped; (4) Toys and Markets. As transversal fields of this research direction, I have detected the clear introduction of robot-based pet therapy [15,16,78,79,80,81,82,83,84,85,86] and the impact of the COVID-19 pandemic on the research activity [17,18]. The latter opened much discussion around the use of SRs in rehabilitation and assistance, complimenting the economic and ethical sphere. Ethical issues have arisen around the key question that SRs cannot provide true selflessness, compassion and warmth, which should be at the heart of an assistance system. Scholars of epistemology are worried that SRs, with increased use, could even increase long-term loneliness, reducing the actual contact people have with humans and increasing a sense of disconnection. This, obviously, is not applicable when SRs are used either as facilitators or mediators among humans, as in most cases in domotics or in some applications in the care of autism, such as the robot Kaspar [88,89,90,91].

It is precisely this role that makes us reflect on the further opportunities of SRs in telerehabilitation applications that can occur in three important sectors:

- As facilitators/mediators to put fragile and/or needy subjects in contact with the health system and/or family members for more complete support of rehabilitation monitoring.
- As support in a more tailored patient-centered therapy by adapting SRs to the patient’s telerehabilitation needs.
- In the domiciliation of care also integrated on the basis of the previous point, with the emerging robotic rehabilitation technologies of the upper and lower limbs integrated into the telerehabilitative pathways and processes.

When we reflect on SRs, and if we are worried about the above-listed problems (increasing loneliness, reducing contacts, etc.), we must also see the flip side of the coin; that is to say that, in this pandemic season, a robot of this type could provide answers to many problems that are encountered in nursing homes and hospitals, such as lack of personnel. In times of lockdown, many elderly and disabled people are left completely alone in their homes and sometimes without adequate health care. Furthermore, even leaving out the COVID-19 pandemic, there was already a problem of assistance (worldwide and in every period) for the elderly, the frail, the disabled, the sick, the lonely and the non self-sufficient. My opinion is that, in general, robotic caregivers should not only be viewed with suspicion but also as a possible opportunity for support. There is no doubt that robotics will be an important part of the health and care of the future. The robots will assist in surgical interventions (in presence or remotely), rehabilitation, in home automation, they will take care of hospital hygiene, dispense lunch and medicines and support of various kinds in general. It is certainly true that robots are not currently able to express the emotions of a human being, however they can do a job in a precise and effective way and could be of great help in dealing with the problems of disability and many problems in health care.

From an economic point of view, it is very interesting for insurance companies under various aspects, ranging from the possibility of developing new insurance formulas that revolve around the use of care-robots, as well as the introduction of new policies that cover the risks of using robots. As for other applications of artificial intelligence, a key point for the diffusion of SRs will clearly be the opinion and the acceptance, the so-called last yard, of all the involved actors, ranging from physicians, nurses and caregivers to patients with their familiars. Therefore, it will be necessary to set up dedicated studies based on dedicated large surveys [92,93] to face the last yard, in which artificial intelligence cannot fail to play a key role [94], given that artificial intelligence will be, for example, fundamental for specifying the level and characteristics of the empathy of social robots in the near future. All this is of basic importance because, according to studies focused on bibliometric indicators, we are witnessing significant growth in this sector. In the study reported in [95], for example, it is documented that the field started growing since the mid-1990s, and after 2006 [95], we can observe a larger amount of publications. The authors [95] obtained academic article data from the robotics and the social robotics fields, highlighting the important increasing number of publications on SRs (a) by number of articles and (b) proportion in relation to all-robotics research. Furthermore, now, official studies show that the social robots market is (https://www.mordorintelligence.com/industry-reports/social_robots_market) [96] estimated to grow at a compound annual growth rate of about 14% over the forecast period 2021 to 2026 thanks to the rise of research in the field of artificial intelligence (AI), natural language processing (NLP) and the development of platforms such as the robotic operating system, which enabled the rise of social robotics.
